# Staphylococcal-Produced Bacteriocins and Antimicrobial Peptides: Their Potential as Alternative Treatments for *Staphylococcus aureus* Infections

**DOI:** 10.3390/antibiotics9020040

**Published:** 2020-01-21

**Authors:** Logan L. Newstead, Katarina Varjonen, Tim Nuttall, Gavin K. Paterson

**Affiliations:** 1 Royal (Dick) School of Veterinary Studies and The Roslin Institute, University of Edinburgh, Easter Bush Campus, Midlothian EH25 9RG, UK; L.Newstead@sms.ed.ac.uk (L.L.N.); Tim.Nuttall@ed.ac.uk (T.N.); 2 AniCura Djursjukhuset Albano, Rinkebyvägen 21A, 182 36 Danderyd, Sweden; katarina.varjonen@anicura.se

**Keywords:** bacteriocins, antimicrobial peptides, *Staphylococcus*, *Staphylococcus aureus*, MRSA

## Abstract

*Staphylococcus aureus* is an important pathogen of both humans and animals, implicated in a wide range of infections. The emergence of antibiotic resistance has resulted in *S*. *aureus* strains that are resistant to almost all available antibiotics, making treatment a clinical challenge. Development of novel antimicrobial approaches is now a priority worldwide. Bacteria produce a range of antimicrobial peptides; the most diverse of these being bacteriocins. Bacteriocins are ribosomally synthesised peptides, displaying potent antimicrobial activity usually against bacteria phylogenetically related to the producer strain. Several bacteriocins have been isolated from commensal coagulase-negative staphylococci, many of which display inhibitory activity against *S. aureus in vitro* and *in vivo*. The ability of these bacteriocins to target biofilm formation and their novel mechanisms of action with efficacy against antibiotic-resistant bacteria make them strong candidates as novel therapeutic antimicrobials. The use of genome-mining tools will help to advance identification and classification of bacteriocins. This review discusses the staphylococcal-derived antimicrobial peptides displaying promise as novel treatments for *S. aureus* infections.

## 1. Introduction

*Staphylococcus aureus* is a frequent opportunistic pathogen of humans and animals that is capable of causing a variety of infections including skin and soft tissue infections, mastitis, urinary tract infections (UTIs), osteomyelitis, meningitis, food poisoning, biofilm-associated infections or septicaemia [1,2,3]. These can range from trivial and self-limiting to severe and life-threatening. *S. aureus* is a leading cause of nosocomial infections, implicated in 30% of infectious endocarditis cases [4,5], and the second most common cause of hospital-acquired pneumonia [6,7]. However, *S. aureus* is also a commensal organism, with 20–30% of humans persistently colonised nasally by the bacteria [8,9,10]. There is an epidemiological link between nasal carriage of *S. aureus* and subsequent infection with the carriage strain, especially among hospitalised individuals [9,11]. Risk factors for *S. aureus* infection include prolonged hospitalisation (especially intensive care), surgery, orthopedic and nursing implants, compromised immunity, skin barrier defects, and inflammatory diseases such as atopic dermatitis. The pathogenicity of *S. aureus* is attributed to an array of virulence factors, which include toxins such as enterotoxins, exfoliative toxins, and Panton-Valentine leucocidin (PVL) [12,13]. *S. aureus* can cause disease in healthy individuals as a result of expression of these virulence factors [1,13]. *S. aureus* also has the ability to form biofilms both on medically implanted devices and on tissue [14]; these characteristics allow *S. aureus* to invade tissue and disseminate, causing systemic disease. The emergence of antimicrobial resistance makes the treatment of *S. aureus* infections a clinical challenge, with many strains displaying methicillin-resistance (MRSA) or multidrug resistance (MDR) [15]. Methicillin-resistance is typically mediated by *mecA*, or less frequently by *mecC*, located on the staphylococcal chromosomal cassette *mec* (SCC*mec*), and is associated with resistance to virtually all β-lactam antibiotics [15,16,17]. Multidrug resistance is typically defined as acquired resistance to three or more classes of antibiotic, with some *S. aureus* strains possessing resistance to all available antibiotics [18]. Topical mupirocin application is often used to eradicate nasal MRSA colonisation pre-operatively to prevent infections, however there are reports of increasing mupirocin resistance [19]. As such, finding alternative treatments for MRSA infections is a public health priority worldwide [20]. 

*S. aureus*, including MRSA, can be isolated from healthy and diseased animals, from companion animals to livestock [21,22,23,24]. *S. aureus* infection has serious welfare implications; some of the most severe infections can be seen in food animals, such as poultry, where the bacteria can cause comb necrosis, chondronecrosis and septicaemia [25,26], and in dairy cattle, where it is one of the causative agents of mastitis [27,28]. *S. aureus* causes chronic, sub-clinical intramammary infection in cattle, resulting in increased somatic cell count in the milk, and as such, decreased milk quality, in addition to decreased milk yield, increased veterinary and labour costs, and loss due to culling [27,28,29]. As a result, bovine mastitis is one of the most economically important diseases in animals. Colonisation and infection in animals also poses a threat to human health, so called livestock-associated MRSA (LA-MRSA), due to the risk of zoonotic transmission, via the food chain or through direct contact [30]. This represents the third recognised epidemiological form of human MRSA along with hospital-associated MRSA (HA-MRSA) and community-associated MRSA (CA-MRSA). Holistic approaches such as improved biosecurity on farms, vaccine development, and selective breeding for animals resistant to pathogens, have yet to succeed in the control of *S. aureus* infections in animals [31,32,33,34,35], increasing the urgent need for the development of novel antimicrobials.

### Bacteriocins as Novel Antimicrobials 

In recent years, the importance of the natural microbiota in health and disease has been highlighted [20,36,37,38,39,40]. In particular, the normal diverse healthy-state microbiota may help regulate inflammation and help prevent colonisation and invasion by potentially pathogenic organisms [41]. One of the ways by which commensal bacteria regulate colonisation by invasive pathogens is via bacteriocin production [20]. Bacteriocins are ribosomally synthesised peptides that display antimicrobial activity against bacteria closely related to the producer strain, but to which the producer strain itself is resistant [42,43]. Bacteriocin resistance genes are typically present concomitantly with bacteriocin structural genes. The mechanisms of resistance include antagonistic bacteriocin receptors or specialised ATP-binding cassette efflux transporters [43,44,45]. As the target strains and producer strains typically share an ecological niche, these specific resistance mechanisms contribute to producer strain survival [43,46,47,48]. Bacteriocin production is an important trait for bacterial fitness, allowing competition against other microorganisms within a niche [49]. However, bacteriocin activity is more complex, with some shown to act as signaling peptides in both quorum sensing systems or interaction with the host immune system [48,50,51]. Some bacteriocins are multifunctional, such as BacSp222 produced by *Staphylococcus pseudintermedius* 222, which features bacteriocin activity, cytotoxicity towards eukaryotic cells and immunomodulating properties [52]. The seeming ubiquity of bacteriocins, despite the energetic costs of production, supports the theory that they are important to bacterial success beyond their role as antimicrobial peptides, and up to 99% of bacteria are thought to produce at least one bacteriocin [53]. Bacteriocins have become an important target in the search for novel antimicrobials as a result of their abundance and activity against a range of pathogens. 

Bacteriocins possess several advantages over traditional antibiotics as a treatment for bacterial infections. Firstly, they typically possess a very narrow spectrum of activity, resulting in less disruption to the microbiota, which can increase susceptibility to pathogenic invasion and has been associated with several inflammatory or metabolic diseases [54]. Narrow spectrum antimicrobials also generate less selective pressure for the development of resistance in non-target organisms [55]. The mechanism of action of bacteriocins is distinct from most antibiotics, meaning they are effective against antibiotic-resistant strains of bacteria [56]. Many bacteriocins are also able to target quiescent cells as well as those actively dividing [57,58]. As ribosomally synthesised peptides, they are amenable targets for bioengineering, and can be modified relatively easily to improve characteristics such as potency, solubility, and stability [56,59]. They also show antimicrobial activity at very low concentrations compared to antibiotics (typically nanomolar concentrations) [46]. As peptides, they are susceptible to digestive enzymes; this improves their safety and minimizes disruption to the gastrointestinal microbiota but might limit them to parenteral or topical administration [54]. Several bacteriocins, such as nisin, have been approved for use as food bio-preservatives and granted generally regarded as safe (GRAS) status [60,61]. However, despite their use in the food industry, it is only recently that attention has been turned to potential use of bacteriocins as alternative antimicrobial therapies. Many *Staphylococcus* species have been shown to produce bacteriocins (Table 1), although bacteriocin production is a strain-specific, not a species-specific, trait [62]. Coagulase-negative *Staphylococcus* spp. (CoNS) are commonly found in the commensal skin microbiota [63]. As bacteriocins typically display antimicrobial activity against strains closely phylogenetically related or within the same niche as the producer, staphylococcal bacteriocins (referred to as staphylococcins) could be promising candidates for the treatment of *S. aureus* infections [64,65]. This review will explore fully and partially characterised staphylococcins, and their therapeutic potential as novel alternatives to traditional antimicrobials in the treatment of *S. aureus* infections. 

## 2. Staphylococcins

A large number of bacteriocins have been isolated from *Staphylococcus* species. *S. aureus* is a prolific bacteriocin producer, with approximately 10 bacteriocins and bacteriocin-like inhibitory substances (BLIS) identified [46,66,67]. Six well-characterised bacteriocins have been isolated from *Staphylococcus epidermidis* [46,64,68,69,70]. Many other CoNS produce bacteriocins, and several have been shown to exert inhibitory activity against *S. aureus*, making them promising candidates for further research. Gram-positive bacterial derived bacteriocins tend to be highly cationic heat stable molecules [71,72]. Staphylococins are most commonly encoded on plasmids or other mobile genetic elements, although they can be chromosomally encoded [51,73]. 

**Figure 1:**
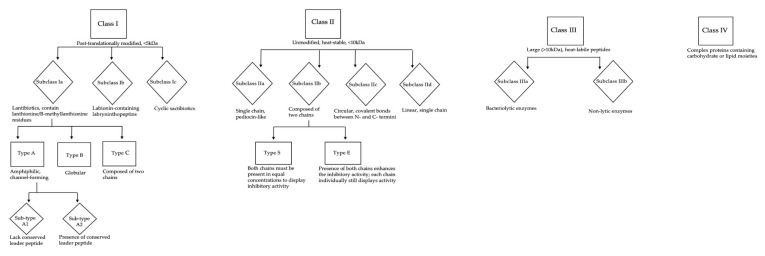
Classification of Gram positive-derived bacteriocins.

Gram-positive and Gram-negative bacteriocins have distinct classification systems; there are four classes of Gram-positive bacteriocins, each containing several sub-classes (Figure 1) [46,74]. The majority of staphylococcins belong to class Ia, also known as lantibiotics. These are small (<5 kDa), post-translationally modified peptides, containing lanthionine or β-methyllanthionine residues [43,71], and possess relatively broad spectrum activity for bacteriocins, typically demonstrating antimicrobial activity against a range of Gram-positive organisms [71]. Lantibiotics are the most extensively studied class of bacteriocins, and as a result, their mechanism of action is relatively well understood. The majority of lantibiotics cause bacterial cell lysis and death via membrane potential-dependent permeabilisation or transmembrane pore formation [51,75,76]. The lantibiotic epidermin and its natural variant gallidermin can also inhibit peptidoglycan biosynthesis by binding membrane-bound lipid II, a peptidoglycan precursor [56,77,78,79]. These bacteriocins bind distinct sites from those targeted by the antibiotic vancomycin, allowing them to maintain efficacy against vancomycin-resistant bacterial strains [80]. The epidermin group of bacteriocins also have the potential to inhibit biofilm formation due to their ability to disrupt teichoic acid biosynthesis [75,81]. Pep5, a bacteriocin produced by *S. epidermidis*, binds negatively charged lipoteichoic acids, initiating autolysis of the target cell due to release and activation of cell wall hydrolysing enzymes [51]. This demonstrates that bacteriocins can inhibit target strains through several mechanisms, both bacteriostatic and bactericidal. 

Subclass Ic, the sactibiotics, are extensively post-translationally modified bacteriocins characterised by the presence of cross-links between the thiol group of cysteine residues and the α-carbon of acceptor amino acids [42,82]. Only a handful of sactibiotics have been characterised, nearly all from *Bacillus* spp. However the first staphylococcal-derived sactibiotic, hyicin 4244, was recently discovered by Freitas De Souza Duarte et al. [83]. Many of the first described sactibiotics were circular, leading to the initial classification of sactibiotics as class IV [84]. However, the existence of linear sactibiotics has resulted in some discrepancy regarding the position of sactibiotics within bacteriocin nomenclature; it has been suggested they represent a novel class, class V, but currently they are tentatively considered a subgroup of class I [84,85]. As more sactibiotics are isolated and characterised, a robust classification of these substances may be elucidated. 

The remaining classes of bacteriocin are not as well characterised as the class I peptides. Class II, compromised of four subclasses, contains fewer members than seen in class I. Most belong to class IId; linear, single chain peptides, typically unmodified and of molecular mass below 10 kDa [66]. Class IIb contains bacteriocins composed of two chains. These bacteriocins can be further defined as type E, where both components show inhibitory activity alone but the presence of both enhances this activity. However, the two class IIb staphylococcins (C55 and aureocin A70 both produced by *S. aureus*) are type S (synergy) meaning that both chains must be present in equimolar proportions for bacteriocin activity [46,72]. 

Class III bacteriocins are large (>10 kDa), heat-labile proteins in two sub-classes; IIIa, the bacteriolytic enzymes, and IIIb, non-lytic enzymes [81,86]. The *in vitro* and *in vivo* efficacy of lysostaphin, a class IIIa metalloprotease, against a range of pathogens has been studied since the 1960s [87]. The catalytic domain of lysostaphin has three distinct functions (a glycylglycine endopeptidase, an endo-B-N-acetyl glucoamidase, and an N-acetyl-muramyl-L-alanine amidase) allowing it to hydrolyse peptidoglycan components, particularly pentaglycine cross-links [88,89]. These are not typically seen in CoNS, making lysostaphin specific for actively growing and quiescent *S. aureus* [88,89,90]. 

Class IV is the final class of staphylococcins. These bacteriocins are poorly characterised and complex proteins, containing carbohydrate or lipid moieties [46,91]. Currently there is only one known staphylococcin in this group; aureocyclicin 4185, isolated from *S. aureus* 4185. This is a cyclic bacteriocin, thought to be cationic, with high hydrophobic residue content. There is little known about its mechanism of action or spectrum of activity [91]. 

Whilst several staphylococcins have been well characterised (Table 1), there are also many other that are only partially characterised with undefined structures, genetics and activities. Few have been tested for antimicrobial activity against pathogens, although some produced by CoNS have shown promising anti-*S. aureus* activity *in vitro* and *in vivo*.

### 2.1. Studies Showing in vitro Inhibitory Activity against Staphylococcus aureus

Multiple techniques have been used to screen bacterial isolates for bacteriocin production *in vitro*. These include spot-on lawn assays where test producer strains are pipetted in small volumes onto the surface of agar plates, which are overlaid with soft agar containing the indicator (target) strain. Well-diffusion assays can be carried out using whole bacteria or, more commonly, cell-free supernatants [74,120]. The limitation of these assays is that they cannot discriminate between inhibitory activity due to bacteriocins or other antimicrobial substances, such as phenol-soluble modulins or organic acids [51]. The use of whole live bacteria also limits the quantitative data that can be obtained, as these assays cannot provide a minimum inhibitory or bactericidal concentration (MIC/MBC). Some studies utilise inhibition zone or density measurements to calculate arbitary units (AU) of inhibition, however these measurements are hard to standardise and are of less value than MICs. Partially purified protein and peptide antimicrobial substances are often tested for stability and activity under different conditions, such as pH, temperature and following proteolytic digestion by enzymes such as proteinase K or trypsin. Proteolysis-associated loss of activity confirms their protein or peptide structure [43]. Based on these results it is then reasonable to presume the antimicrobial substance is a bacteriocin-like inhibitory substance (BLIS), however, molecular and genomic analysis should be carried out to confirm the molecule is a bacteriocin and further characterise and classify it. 

The lantibiotics Pep5 and epidermin, both produced by *Staphylococcus epidermidis*, were shown to inhibit 14 and 13 of 16 test strains of *S. aureus*, respectively, including the endemic Brazilian MRSA clone A/22C. Pep5 also inhibited a mupirocin-resistant strain [74]. Further studies showed that Pep5 inhibited 63% and epidermin 87% of 165 *S. aureus* isolates from bovine mastitis cases in South America [85]. Hyicin 3682 from *S. hyicus*, a member of the epidermin-like group, inhibited 15 of 16 *S. aureus* test strains [99,100]. Hominicin from *S. hominis* displayed potent activity against multiple strains including *S. aureus* ATCC 25923, MRSA ATCC 11435, and vancomycin-intermediate *S. aureus* CCARM 3501 [97,98], at MICs of 0.06μg/mL, 0.96 μg/mL, and 3.82 μg/mL, respectively [98]. BacCh91, produced by *S. aureus* CH91, inhibited four test strains of *S. aureus* (ATCC25293, Newman, M-122 and RN4220), with an MIC of 4.0–6.0 μM [92]. Gallidermin, isolated from poultry-associated *Staphylococcus gallinarum*, has been shown to be bactericidal against both MRSA and methicillin-sensitive *S. aureus* (MSSA) [96,121]. Gallidermin demonstrated both an MIC and MBC of 1.25 μg/mL against MSSA, and 1.56 μg/mL against MRSA [96]. Gallidermin was also able to inhibit biofilm formation of *S. aureus* SA113 at 0.16× the MIC [75]. Biofilm inhibition by gallidermin is due to repression of biofilm related genes *atl* and *ica*, encoding autolysin and polysaccharide intercellular adhesin (PIA), respectively. These gene products are involved in attachment to surfaces and cell aggregation, important steps in biofilm formation. However, gallidermin was not as effective against pre-formed biofilms, requiring 8× MIC to display inhibitory activity with 0.1–1.0% ‘persister’ cells still remaining [75]. The activity against planktonic cells and biofilm formation at low concentrations combined with the absence of cytotoxicity against fibroblasts or peripheral blood mononuclear cells, suggests gallidermin is a promising candidate as a therapeutic antimicrobial agent.

Recently, a natural variant of the lantibiotic nisin (nisin J) was isolated from *Staphylococcus capitis* APC2923 [122]. Nisin is a well-characterised bacteriocin first isolated from *Lactococcus lactis*; there are now at least ten known natural nisin variants produced by various *Lactococcus*, *Streptococcus* and *Blautia* spp. [123,124,125,126,127,128]. Nisin J appears resemble streptococcal nisin variants more closely than lactococcal variants [122]. Nisin J inhibited staphylococcal isolates, including *S. aureus*, with greater efficacy than nisin A or Z [122]. Like other nisin variants, the nisin J-encoding gene cluster resides on a plasmid; this has led to the suggestion that the gene cluster has been acquired via horizontal gene transfer, possibly explaining why nisin variants are isolated from several species [122].

A bacteriocin produced by *Staphylococcus hyicus* 4244 (hyicin 4244) was shown to have inhibitory activity against other staphylococcal species [83]. This bacteriocin inhibited ten clinical *S. aureus* isolates from humans and cattle, and demonstrated efficacy against MRSA and MDR strains. Hyicin 4244 also showed potential as an *S. aureus* biofilm-inhibiting agent. Genome analysis and further characterisation showed it belonged to class Ic, the sactibiotics [103], the first staphylococcin in this subclass. 

BacSp222 is a class II staphylococcin produced by *S. pseudintermedius* [52]. This tryptophan residue rich bacteriocin showed no resemblance in peptide sequence to other bacteriocins beyond limited similarities to class II bacteriocins such as epidermicin NI01 and lacticin Q and Z. BacSp222 inhibited four *S. aureus* test strains, including MRSA and *S. aureus* CH91, the producer strain of bacteriocin BacCH91, with an MIC of 0.89–1.30 μM. BacSp222 possesses some unusual characteristics for a bacteriocin; it is resistant to protease digestion and is active against the producer strain, although the MIC required (2.1 μM) was much higher than the MIC against a non-producer *S. pseudintermedius* strain (0.16 μM). Capidermicin and epidermicin NI01 are also class II bacteriocins, both belonging to the aureocin A53-subgroup [64,105]. Capidermicin inhibited all four test strains of *S. aureus* (NCDO1499, DPC5297, Newman, and RF122) with an MIC of 3.1–10 μg/mL, as well as *S. pseudintermedius* (MIC 10 μg/mL) [105]. Epidermicin NI01 inhibited MRSA *in vitro* and was not toxic to erythrocytes or dermal fibroblasts, even at a concentration of 100× the MIC [64], making epidermicin NI01 a promising candidate treatment for *S. aureus* and *S. pseudintermedius* infections. The latter being a prominent pathogen in companion dogs, particularly in pyoderma [129], with antimicrobial resistance, including methicillin-resistance and MDR isolates presenting a challenge to treatment [130,131,132]. As with *S. aureus*, bacteriocins and related products may have a valuable role in new approaches to tackle this pathogen.

Many studies have demonstrated the efficacy of lysostaphin against *S. aureus*. Zygmunt et al. [114] showed lysostaphin inhibited 16 MRSA isolates with 4–8× the potency of synthetic β-lactams. Lysostaphin inhibited 111 clinical MRSA isolates in a study by Huber and Huber [116], with a subsequent study by von Eiff et al. [90] showing inhibition of 429 MRSA and MSSA strains, isolated from both commensal nasal swabs and cases of bacteraemia. Lysostaphin was also shown to kill biofilm-associated *S. aureus* cells and disrupt the biofilm extracellular matrix [115]. Catheters coated with lysostaphin showed complete clearance of *S. aureus* compared to control catheters, where an average of 493 CFU were recovered. The inhibitory activity of lysostaphin was maintained on the catheters for at least four days post-coating, suggesting lysostaphin is able to bind to plastic surfaces and retain anti-staphylococcal activity for several days [111]. Due to this, lysostaphin has potential use as a preventative and treatment for biofilm-associated infections. The promising inhibitory activity of lysostaphin against *S. aureus* led to investigations of its efficacy *in vivo*. 

### 2.2. Models of in vivo Bacteriocin Therapy for Staphylococcus aureus Infection 

Animals are often used as models of human disease to determine the safety and efficacy of treatments under physiological conditions [133]. Lysostaphin has been widely tested in a range of *in vivo* systems. A single intravenous injection of lysostaphin decreased *S. aureus* bacterial load and increased survival rates in rodent models of infection, including mastitis, peritonitis and sepsis [117,118,119]. In mouse models of renal disease, a single intravenous dose of lysostaphin (from 1.56 mg/kg to 50 mg/kg) significantly reduced viable *S. aureus* bacterial counts from renal lesions by 95% compared to an untreated control [112], whilst another study showed a 39–78% reduction in *S. aureus* bacterial burden and a 55–65% decrease in mortality, dependent on the dose [113]. Rabbit models were used to test the efficacy of lysostaphin against *S. aureus* associated aortic valve endocarditis [88,109]. Both studies demonstrated a reduction in *S. aureus* counts following administration of lysostaphin, with a single dose showing a 3.7–6.63 log_10_ and 7.27–8.5 log_10_ CFU/g decrease in bacterial counts compared to antibiotic-treated and untreated controls, respectively [88,109]. In one case, this result was seen three days post-treatment [109] whilst the second study noted that by 30 h post-treatment there was no difference in *S. aureus* counts between treated and control animals [88]. It is possible that the choice of vehicle and route of administration affects the duration of lysostaphin activity *in vivo*; this was further highlighted in a cotton rat model of *S. aureus* colonisation of the nares, where 0.5% lysostaphin in a petroleum-based vehicle eradicated MRSA and MSSA in 93% of subjects whilst lysostaphin in a PBS solution resulted in eradication in only 33% [57]. There has been a single trial of lysostaphin to eradicate nasal colonisation in humans comparing three treatment groups with an intranasal spray of 0.5% lysostaphin in saline 3 × daily for five days, intranasal neomycin/polymyxin B/bacitracin spray 3 × daily for five days, or no treatment [110]. 40% of the lysostaphin-treated group were cleared of *S. aureus* colonisation, compared to 6% of the antibiotic-treated and 3% of the untreated group. The effect of lysostaphin appeared to be transient, however, with re-colonisation seen by Day 11. It is possible that if delivered in a different vehicle, a longer-lived effect may be seen and this is a promising direction for further studies. 

A relatively new model has been introduced for first-line *in vivo* testing, using *Galleria mellonella* (greater wax moth) larvae. These are an alternative to mammalian models as their immune system shows a high degree of structural and functional similarities to mammals [134]. These models are more accessible, inexpensive and ethical than using experimental mammals [106]. This model was used to test the efficacy and safety of epidermicin NI01 for *S. aureus* infection, which was non-toxic to the larvae and increased survival compared to untreated controls [106]. However, no quantified data for *S. aureus* bacterial burden before and after treatment was provided, which would be helpful in understanding its efficacy as an antimicrobial agent. Epidermicin NI01 was also tested in a cotton rat model of *S. aureus* nasal colonisation [107]; the nares of cotton rats structurally resemble those of humans, making it a useful model [135]. Subjects were treated with a single dose of 0.8% epidermicin NI01, twice daily treatment for three days with 0.04% epidermicin NI01, 0.2% epidermicin NI01, 2% mupirocin, or a vehicle control. A single dose of 0.8% epidermicin NI01 was most effective, resulting in a significant reduction in nasal MRSA burden and eradication in three of five test subjects [107]. Epidermicin NI01 is therefore a potential novel therapeutic for *S. aureus* nasal colonisation. 

## 3. Other Antimicrobial Substances with Anti-*Staphylococcus aureus* Activity 

### 3.1. Bacteriocin-Like Inhibitory Substances

A partially purified antimicrobial substance has been derived from *Staphylococcus pasteuri* RSP-1 [65]. Cell-free supernatant (CFS) from *S. pasteuri* RSP-1 was found to inhibit 11 out of 14 *S. aureus* test strains [65]. Live-dead assays suggest this substance is bactericidal, causing membrane damage and subsequent cell death in target cells. Antimicrobial activity of the CFS was lost following proteolytic digestion, whilst nuclease, amylase and lipase had no effect, confirming the substance is proteinaceous. It was heat stable up to 121 °C and at a range of pH, although a gradual loss of activity was seen with increasing pH. These characteristics are suggestive of a bacteriocin. The substance, named pasteuricin, has a molecular weight of 5 kDa [65], suggesting it belongs to bacteriocin class I or II, but further characterisation is needed.

*Staphylococcus capitis* TE8 isolated from the skin microbiota of humans showed antimicrobial activity against a range of Gram-positive organisms, including *S. aureus*, but had no effect on Gram-negative organisms [136]. Partially purified CFS extract also demonstrated this activity, which was lost with proteinase K digestion, suggesting the inhibitory effect was mediated by production of a BLIS. Genomic analysis revealed *S. capitis* TE8 possesses multiple antimicrobial peptide (AMP) gene clusters, including those encoding an epidermicin-like peptide, a gallidermin-like peptide, and several phenol-soluble modulins [136]. The epidermicin-like peptide seen may be capidermicin, an epidermicin variant recently isolated from *S. capitis* CIT060 [105]. However, it is possible that the BLIS and gene-clusters possessed by *S. capitis* TE8 are novel bacteriocins. 

Nakatsuji et al. [62] explored the abundance of AMP production in the skin microbiota of humans with atopic dermatitis (AD) and healthy controls; they found that AMPs were common in the microbial communities of healthy subjects, but not those with AD. The application of AMP-producing *S. hominis* or *S. epidermidis* to the skin of AD subjects significantly decreased *S. aureus* burden on the skin compared to untreated and vehicle-treated controls, supporting the protective role of AMP-producing CoNS within the skin microbiota. Further investigation of commensal CoNS isolates revealed a strain of *S. hominis* (A9) with potent antimicrobial activity against *S. aureus*. Application of *S. hominis* A9 to sanitised pig skin coated with 1 × 10^5^ CFU/cm^2^ *S. aureus* or to mice colonised with *S. aureus* significantly decreased *S. aureus* counts, with application twice daily for one week eliminating *S. aureus* colonisation in the mouse model. In contrast, application of killed *S. hominis* A9 or a non-inhibitory control strain of *S. hominis* had no effect. Genomic and biochemical analysis revealed *S. hominis* A9 produces two AMPs, predicted to be lantibiotics based their on structure and amino acid composition. These AMPs, named *Sh*-lantibiotic-α and *Sh*-lantibiotic-β, were encoded within a gene cluster containing *lanM*, *lanC*, and *lanT* homologs. These genes were not detected in non-inhibitory *S. hominis* strains. Purified *Sh*-lantibiotic-α and *Sh*-lantibiotic-β inhibited *S. aureus* on sanitised pig skin at a concentration of 0.5 nM, whilst concentrations up to 10 nM had no effect on *S. hominis* A9, the producer strain. *Sh*-lantibiotic-α and *Sh*-lantibiotic-β were able to suppress clinical *S. aureus* isolates, including MRSA USA300, but had no effect on commensal species isolated from the skin such as *Propionibacterium acnes*, *S. epidermidis*, and *Corynebacterium minutissimim*. This potent anti-*S. aureus* activity with limited disruption to microbiota make *Sh*-lantibiotic-α and *Sh*-lantibiotic-β promising candidates for further development as novel therapeutics for *S. aureus* infection in AD and other skin conditions. 

### 3.2. Inhibitory Staphylococcal Strains 

Several strains of CoNS inhibit *S. aureus* in agar-based antagonism assays. Although the antimicrobial substances responsible have not been isolated, most are presumed to be BLIS. *Staphylococcus succinus* AAS2 CFS potently inhibited *S. aureus* in well-diffusion assays [137]. Another study [138] found that 28 of 243 *Staphylococcus* isolates produced antimicrobial substances; all were susceptible to proteolytic digestion and thus classified as BLIS. BLIS-producing isolates included *S. chromogenes*, *S. epidermidis*, *S. haemolyticus*, *S. pseudintermedius*, *S. aureus*, and *S. agnetis*. All the BLIS-producing isolates harboured *nukA* or *bsaA2* genes, suggesting these BLIS are related to nukacin ISK-1 or Bsa (a member of the epidermin-like lantibiotics). Purification, classification, and further testing of the inhibitory activity against *S. aureus* is needed to determine their potential as anti-*S. aureus* agents. 77 of 89 *Staphylococcus* isolates from nasal swabs of 37 human volunteers were shown to have inhibitory activity [139]. These isolates belonged to six species: *S. epidermidis*, *S. aureus*, *S. hominis*, *S. lugdunensis*, *S. warneri*, and *S. capitis*. Only two of the total 77 strains, however, showed inhibitory activity against *S. aureus*. 96% of the *S. epidermidis* strains produced BLIS, but these were not further investigated to determine if they were novel or one of the already isolated bacteriocins from this species. A recent study demonstrated AMP production by 21 CoNS strains, belonging to five species; *S. capitis*, *S. hominis*, *S. simulans* and *S. warneri* [20]. Of these, four *S. warneri* strains and one *S. hominis* strain were able to inhibit *S. aureus* [20]. Two strains belonging to *S. capitis*, APC2934 and APC2918, were able to inhibit both *S. aureus* and MRSA test strains [20]. These strains did not possess the structural genes encoding nisin J and colony mass spectrometry did not match the peptides produced by these *S. capitis* strains to any listed on BACTIBASE [20], suggesting these are potentially novel bacteriocins.

Carson et al. [140] investigated 441 non-aureus staphylococci (NAS) isolates; 40 of the isolates showed inhibitory activity against a bovine mastitis *S. aureus* strain; of these, 23 also inhibited MRSA. These strains belonged to *S. capitis*, *S. chromogenes*, *S. epidermidis*, *S. pasteuri*, *S. simulans* and *S. xylosus*. Only five of these species inhibited *S. aureus* in well-diffusion assays using chloroform-extracted cell-free supernatant; all five supernatants were inactivated by proteinase K, suggesting the active components are BLIS secreted by the bacteria. The genomes of the 441 NAS were studied for the presence of bacteriocin biosynthetic gene clusters. 105 clusters were identified from 95 NAS isolates, belonging to 16 species (Table 2), but there was no obvious clustering based on phylogeny or bacteriocin class. Ten of the NAS genomes encoded two clusters, belonging to different classes, suggesting these isolates have the potential to produce two bacteriocins [140]. This data shows that the 95 isolates possessing bacteriocin gene clusters have the potential to produce bacteriocins. However, only 40 of the isolates displayed inhibitory activity *in vitro*. The discrepancy between presence of bacteriocin genes and production of bacteriocins is likely due to the influence of growth conditions on bacteriocin production; the availability of nutrients, presence of stressors, temperature, and choice of media can all affect bacteriocin production [20]. This highlights the importance of screening methods when trying to identify bacteriocin-producing bacteria, suggesting that there may be many more strains capable of producing bacteriocins that have not yet been discovered. Genome-mining tools, such as antiSMASH and BAGEL [141,142], are able to identify bacteriocin gene clusters in bacterial genomes, highlighting those harbouring the potential to produce bacteriocins. These techniques will be invaluable in the search for novel bacteriocins especially as the availability of sequenced genomes increases. 

### 3.3. Staphylococcal-Produced Antimicrobial Substances 

*Staphylococcus* species produce a range of other secretory-AMPs (sAMPs) alongside bacteriocins, and several of these non-bacteriocin AMPs show promise as therapeutic agents for *S. aureus* infections. Esp is a serine protease produced by some *S. epidermidis* strains [143]. It was noted that presence of certain *S. epidermidis* strains within the nasal cavity appeared to influence *S. aureus* nasal colonisation. The CFS of these strains inhibited *S. aureus in vitro*, leading to the purification and identification of Esp. Application of purified Esp or Esp-producing *S. epidermidis* to the nasal cavities of *S. aureus* carriers eliminated *S. aureus* colonisation. Esp is effective against *S. aureus* biofilms, cleaving autolysin-derived murein hydrolases [144] and preventing the release of DNA, one of the structural components of *S. aureus* biofilm extracellular matrices [145,146]. Esp also targets *S. aureus* surface proteins, disrupting host-pathogen interactions [147], allowing Esp to be active against biofilm-forming and planktonic *S. aureus* cells. This suggests Esp could be a very promising antimicrobial agent. 

Lugdunin is a novel antimicrobial, isolated from *S. lugdunensis* IVK28 [148]. It is only produced under iron-limiting conditions on solid agar, again highlighting the importance of growth conditions of producer strains when isolating antimicrobial substances. Lugdunin was encoded by all the *S. lugdunensis* strains analysed, suggesting production is species specific rather than strain specific [148]. Lugdunin is a complex, non-ribosomally synthesised peptide, containing a tryptophan moiety, with no resemblance to any known antimicrobial substances [148]. Lugdunin became the founding member of a new class of antibiotics, the thiazolidine-containing peptide antibiotics. It is suggested that it exerts its antimicrobial activity by depleting bacterial energy resources [148]. Lugdunin demonstrated potent inhibitory activity against a range of Gram-positive organisms, including MRSA and glycopeptide-intermediate *S. aureus* [148]. This antibiotic displayed no toxicity towards human neutrophils or erythrocytes, and retained activity in human serum. Lugdunin was also able to reduce or eradicate *S. aureus* in a mouse model [148]. Together these examples demonstrate the range of antimicrobial substances produced by commensal staphylococci, and their potential as novel treatments for *S. aureus* infection. It is highly likely more remain to be discovered. 

## 4. Conclusions and Future Directions

The commensal bacteria residing in the microbiota play a vital role in protecting the host from invasion of pathogenic organisms. This protective activity is often mediated by bacteriocins, which are ribosomally synthesised peptides produced by bacteria that possess antimicrobial activity. Bacteriocins may be a valuable tool in the future fight against antimicrobial-resistant pathogens due to their novel mechanisms of action, narrow spectrum of activity, and ability to be bioengineered to improve specific qualities desirable in biopharmaceutical agents. Many bacteriocins produced by staphylococci display potent activity against S. aureus *in vitro*; however, the lack of cytotoxicity testing of many bacteriocins is a limitation when assessing their therapeutic usefulness. Although many bacteriocins demonstrate cytotoxicity against eukaryotic cells, often in a dose-dependent manner, this does not entirely eliminate their potential as candidates for treatment of *S. aureus* infections including those caused by methicillin-resistant strains in humans and animals. Thorough evaluation of potential cytotoxic effects and pharmacodynamics of the substance, weighed against its efficacy, is required to determine suitability as an anti-*S. aureus* agent. Genome-mining techniques will facilitate the search for bacteriocin-producing bacterial strains, overcoming some of the limitations of agar assay-based methods, and helping eliminate some discrepancy in the classification of these substances. 

Elucidation of the mechanisms of action of bacteriocins, especially those belonging to classes II-IV, alongside further testing of their efficacy under physiological conditions is required to determine their suitability for therapeutic use. It is important to note that although resistance among target strains to these peptides has yet to be witnessed under laboratory conditions, resistance mechanisms are widespread in producer strains. Therefore, prudence must be exercised if and when bacteriocins and related products are utilised clinically to avoid the spread of resistance and loss of efficacy.

## Figures and Tables

**Table 1:** Well-characterised bacteriocins isolated from *Staphylococcus* species.

Class	Subclass	Subtype	Bacteriocin	Producing Strain	Inhibits *S. aureus:* Strain (MIC)	*in vivo* Model	References
I	Ia	A1	BacCh91 *	*S. aureus* CH9/DSM26258	*in vitro*: ATCC25923, Newman, M-122, RN4220 (4.0–6.0 μM)		[92]
			Epicidin 280 *	*S. epidermidi*s BN280			[70]
			Epidermin *	*S. epidermidis* Tü3298	*in vitro*		[74,93]
			Epilancin 15X *	*S. epidermidis* 15X154			[94]
			Epilancin K7 *	*S. epidermidis* K7			[69]
			Gallidermin *	*S. gallinarum* F16/P57 Tü3298	*in vitro*: ATCC29213, CCUG35601 (1.25–8.0 μg/mL)		[75,95,96]
			Hominicin	*S. hominis* MBBL2–9	*in vitro*: ATCC25923, ATCC11435, CCAR M3501 (0.06–3.82 μg/mL)		[97,98]
			Hyicin 3682	*S. hyicus* 3682	*in vitro*		[99,100]
			Nisin J	*S. capitis APC2923*	*in vitro*		[20]
			Pep5	*S.* *epidermidis 5*	*in vitro*		[73,74,93]
		A2	Nukacin ISK-1 **	*S. warneri* Nukadoko/*S. simulans* 3299			[101]
			Warnericin RB4	*S. warneri* RB4			[102]
	Ic		Hyicin 4244	*S. hyicus* 4244	*in vitro*		[83,103]
II	IIb	S	Aureocin A70	*S. aureus* A70			[104]
		S	C55 *	*S. aureus* C55			[67]
	IId		Aureocin A53 *	*S. aureus* A53			[66,74]
			BacSp222 **	*S.* *pseudintermedius*	*in vitro:* DSM26258, MRSA USA300, KB/8568, ATCC25923 (0.89–1.30 μM)		[52]
II	IId		Capidermicin	*S. capitis* CIT060	*in vitro:* NCDO1499, DPC5297, Newman, RF122 (3.1–10 μg/mL)		[105]
			Epidermicin NI01 *	*S. epidermidis* 224	*in vitro:* 1195, MRSA s37, MRSA s41, MRSA s71 (1.0–2.0 μg/mL)	greater wax moth, cotton rat	[64,106,107]
III	IIIa		Endopeptidase ALE-1 ^†^	*S. capitis* EPk1			[108]
			Lysostaphin **	*S. simulans* biovar Staphylolyticus ATCC1362	*in vitro* (0.002–100 μg/mL)	rat, mouse, cotton rat, rabbit, human	[87,88,90,109,110,111,112,113,114,115,116,117,118,119]
IV			Aureocyclicin 4185	*S. aureus* 4185			[91]

Chemical structure available from: * https://www.bactibase.hammamilab.org; ** https://www.ncbi.nlm.nih.gov/Structure; **†** https://www.rcsb.org; all other chemical structures available from references stylised in bold.

**Table 2:** Strains of *Staphylococcus* found to harbour bacteriocin gene-clusters from 441 non-aureus *Staphylococcus* isolates analysed, the number of isolates possessing bacteriocin production genes that displayed inhibitory activity, and the number of isolates displaying in vitro inhibitory activity against *S. aureus* strains isolated from bovine mastitis cases. Each cluster encodes one bacteriocin [140].

		Class I		Class II
	Lantibiotics	Sactibiotics	Lasso Peptides	
**Number of Bacteriocin Clusters Identified**	29	3	4	69
**Number of Isolates that the Clusters are Present in**	29	3	2	68
**The Species that the Clusters are Present in**	*S. capitis*	*S. capitis*	*S. fleurettii*	*S. equorum*
	*S. chromogenes*		*S. sciuri*	*S. gallinarum*
	*S. cohnii*			*S. haemolyticus*
	*S. epidermidis*			*S. hyicus*
	*S. equorum*			*S. saprophyticus*
	*S. gallinarum*			*S. sciuri*
	*S. sciuri*			*S. simulans*
	*S. simulans*			*S. succinus*
	*S. succinus*			*S. warneri*
	*S. vitulinus*			*S. xylosus*
**Number of Isolates Showing Inhibitory Activity *in vitro***	15	2	1	9
